# Donor-Acceptor-Based Organic Polymer Semiconductor Materials to Achieve High Hole Mobility in Organic Field-Effect Transistors

**DOI:** 10.3390/polym15183713

**Published:** 2023-09-09

**Authors:** Shiwei Ren, Zhuoer Wang, Wenqing Zhang, Yubing Ding, Zhengran Yi

**Affiliations:** 1 Zhuhai-Fudan Innovation Research Institute, Hengqin 519000, China; 2 Faculty of Chemistry and Chemical Engineering, Shandong University, Jinan 250101, China; 3 Key Laboratory of Organic Solids, National Chemical Research Institute of the Chinese Academy of Sciences, Beijing 100190, China

**Keywords:** p-type semiconductor materials, conjugated polymer, Stille coupling, OFET device, hole mobility

## Abstract

Organic polymer semiconductor materials are conveniently tuned to energy levels because of their good chemically modifiable properties, thus enhancing their carrier transport capabilities. Here, we have designed and prepared a polymer with a donor-acceptor structure and tested its potential as a p-type material for organic field-effect transistor (OFET) applications using a solution-processing method. The conjugated polymers, obtained via the polymerization of the two monomers relying on the Stille coupling reaction, possess extremely high molecular weights and thermodynamic stability. Theoretical-based calculations show that PDPP-2S-Se has superior planarity, which is favorable for carrier transport within the main chain. Photophysical and electrochemical measurements systematically investigated the properties of the material and the energy levels with respect to the theoretical values. The maximum hole mobility of the PDPP-2S-Se-based OFET device is 0.59 cm^2^ V^−1^ s^−1^, which makes it a useful material for potential organic electronics applications.

## 1. Introduction

The 2000 Nobel Prize in Chemistry was awarded to three scientists who made outstanding contributions to the field of organic electronics because their research fundamentally changed the way organic polymers were viewed [1,2]. After two decades of development, organic polymer materials have changed from insulating “plastics” to conductive (semi-conductive) functional materials. Generally speaking, the field of organic electronics has been divided into the study of small molecules and macromolecules based on the size of the molecular weight of the organic materials [3,4]. Small-molecule materials have a number of advantages: multiple synthetic routes to the target product, ease of functionalization and modification, uncomplicated purification methods, and easy characterization of the product structure. However, in the process from material to device, small-molecule materials have poor film-forming properties due to easy crystallization and precipitation [5,6]. This has limited the large-scale application of organic semiconductor materials based on small molecules. Macromolecules, also known as polymers, are semiconductor materials with superior film-forming properties and processability, which are advantageous for inkjet printing and high-volume preparation [7,8]. In addition, compared with inorganic semiconductor materials, organic polymer semiconductor materials have the advantages of low costs, flexibility, easy structural modification, and light weight [9]. Therefore, they are potential materials for the preparation of OFETs devices.

**Figure 1:**
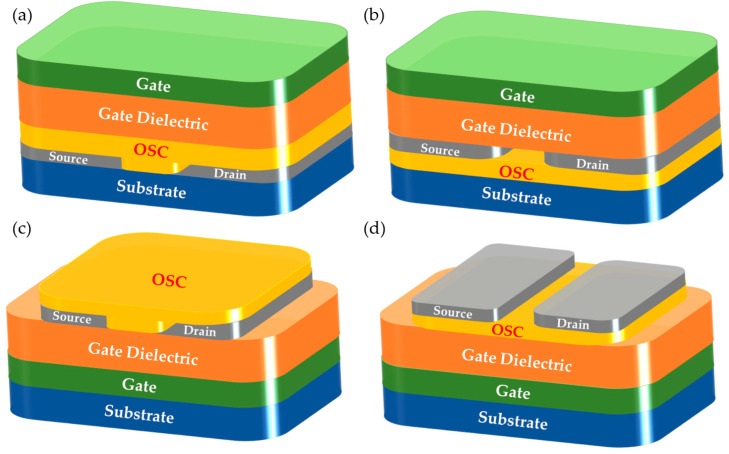
The four configurations of the OFET: (**a**) TGBC; (**b**) TGTC; (**c**) BGBC; and (**d**) BGTC.

OFETs are mainly used as basic logic circuits to transfer holes or electrons. Depending on the structure, they are often classified into four configurations. The top-gate bottom-contact (TGBC) and top-gate top-contact (TGTC) configurations are as shown at the top of Figure 1; bottom-gate bottom-contact (BGBC) and bottom-gate top-contact (BGTC) are illustrated on the bottom of Figure 1 [10]. The organic semiconductor (OSC) layer is the most important component, and its nature strongly controls the overall performance of the device. The performance of the device is generally defined in terms of charge carrier mobility, threshold voltage, and switching ratio, with mobility being the most critical parameter. For applications based on OFETs as the basic component structure, such as organic electrochemical transistors, organic thermoelectric applications, radio-frequency identification tags, and chemical sensors, the mobility needs to satisfy between 0.01 and 1 cm^2^ V^−1^ s^−1^ [11,12,13,14,15]. The p-type, n-type, and ambipolar OSCs are defined and are responsible for transporting holes, electrons, and holes/electrons, respectively. In general, the type of transport function of an OFET device depends mainly on its frontal orbital energy level, i.e., the highest occupied molecular orbital (HOMO) and lowest unoccupied molecular orbital (LUMO) energy levels, and the functional relationship between this energy level and the contact electrode materials (e.g., gold, silver, or aluminum). For p-type organic semiconductors, HOMO levels above −5.50 eV (versus a vacuum level of 0 eV) are generally preferred for the injection and transport of positively charged holes. In the case of n-type OSC materials, a LUMO energy level close to −4.00 eV is favorable for stable electron transport [16,17,18,19]. The HOMO energy levels of materials are mostly controlled by electron-donating groups such as thiophene, benzene, furan, benzodithiophene, dithiophene, etc., whereas the LUMO energy levels of the materials are mostly controlled by electron-acceptor moieties. Therefore, the development of p-type and n-type materials can be based on an all-donor-type structure, an all-acceptor-type structure, as well as a donor-acceptor-type architecture. Here, we adopt the design idea based on the donor-acceptor structure to develop new polymers, where the acceptor is diketopyrrolopyrrole (DPP) and the donors are bithiophene and selenophene, respectively. The “tri-donor strategy” is employed mainly to increase the HOMO level of the polymers, which will lead to an improvement in the hole mobility of the device.

## 2. Materials and Methods

Materials: All chemicals (2-cyanothiophene, potassium carbonate, 11-(3-bromopropyl) henicosane, N-bromosuccinimide, triethyl phosphite, 2,5-bis(trimethylstannyl) selenophene), catalysts (tris (Dibenzylidenacetone)palladiu), and organic solvents (chloroform, chlorobenzene, methanol, hexane, acetone, ethyl acetate, dichloromethane, dimethylformamide) were purchased from Aladdin and Sigma-Aldrich, which were used as received. DPP-2S was synthesized referencing the previous literature [20]. Details of specific synthetic routes and purification methods for subsequent small molecules as well as polymers are presented in the Supplementary Information (SI).

Characterization: The nuclear magnetic resonance (NMR) spectrum of small molecules was measured in deuterated chloroform as solvent (Bruker AVANCE 400, Mannheim, Germany). The mass spectrometry was performed on an HRMS spectrometer (Thermo Scientific Q Exactive, Waltham, MA, USA). The molecular weight (Mp, Mn, Mw, Mv) of PDPP-2S-Se was estimated with high-temperature (150 °C) gel permeation chromatography (Agilent PL-GPC 220, Denver, CO, USA). The infrared spectra of the monomers and polymers were carried out on a Thermo Scientific iN10 (Waltham, MA, USA) spectrometer. Thermodynamic stability of PDPP-2S-Se was measured using thermogravimetry (TGA) and differential scanning calorimetry (DSC) in a nitrogen atmosphere (HITACHI STA200, Hitachi, Japan). The elemental analysis of PDPP-2S-Se was measured in CNHS mode (Thermo FLASH2000, Waltham, MA, USA). Absorption spectra were recorded on a UV-Vis-NIR spectrophotometer (Thermo Evolution 350, Waltham, MA, USA) using a 1 cm wide standard quartz cell and on a quartz chip (45 mm × 12 mm × 1 mm), respectively. Electrochemical measurements were performed using cyclic voltammetry in acetonitrile solutions containing tetrabutylammonium hexafluorophosphate (TBAPF_6_) (CHI760E, CH Instruments, Bee Cave, TX, USA) with a scan rate of 0.05 V s^−1^. Ag/AgCl electrodes, Pt electrodes, as well as glassy carbon electrodes were, respectively, used as reference electrodes, counter electrodes, and working electrodes. Gaussian 16 program using B3LYP hybrid density functional and D3/def2tzvp basis set were used for the Density Functional Theory calculations. The optimized geometry and spatial coordinates based on methyl-substituted trimers are shown in Figure S3 and Table 1, respectively.

OFET Device: The substrates were ultrasonically cleaned sequentially with deionized H_2_O, acetone, and isopropyl alcohol. The rinsed substrates were dried before being processed with Plasma (MiniFlecto, Plasma Technology GmbH, Herrenberg, Germany). Prior to the deposition of the polymer, the SiO_2_ gate dielectrics were treated with octadecyl-trichlorosilane (OTS) in vacuum. Electrical mobility characteristics were obtained using a Keithley 4200 SCS (Tektronix, Beaverton, OR, USA) analyzer in a glove box. To optimize device performance, various OFET device channel lengths were selected for the identical channel width condition (1400 µm). The optimum mobility was observed at a channel length of 30 µm.

## 3. Results

### 3.1. Synthesis

**Figure 2:**
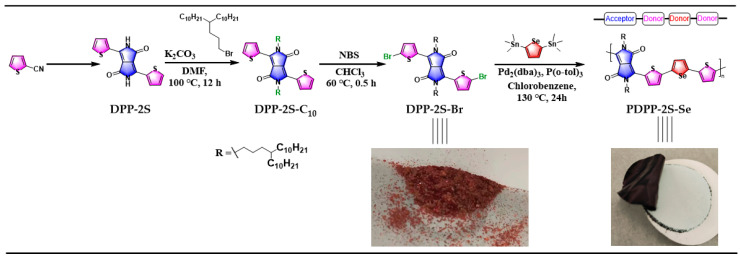
Synthesis of DPP-based small molecule and PDPP-2S-2Se copolymer.

The synthetic route to the polymer PDPP-2S-Se obtained based on Stille coupling polymerization is shown in Figure 2. Commercially available 2-cyanothiophene was used as a precursor for the synthesis of DPP-2S in high yields. The alkylation reaction was further utilized to conveniently introduce an aliphatic chain at the N-position, which in turn improved the solution processability of the material [21]. The reaction of DPP-2S-C10 with a stoichiometric equivalent of NBS reagent (2.05 eq) allowed for the introduction of bromine atoms at position 2 of the left and right thiophene rings. It is worth mentioning that this step is generally carried out under ambient conditions, which typically takes more than 8 h and always relies on thin-layer chromatography detection to ensure the progress of the reaction [22,23,24]. The adoption of heated conditions reduced the reaction time to 0.5 h with high yields. Similarly, the use of catalytic amounts of acid, such as hydrochloric or acetic acid, can significantly minimize the reaction time for this reaction. After purification with column chromatography, the monomer DPP-2S-Br was further purified via reprecipitation, which was performed to ensure that the quality of the monomer was pure enough. DPP-2S-Br exhibits extremely poor solubility in methanol, and the product obtained after reprecipitation using methanol is also shown in the figure below. The polymerization of the two monomers, containing -Br and -Sn, respectively, was catalyzed by Pd (0) in chlorobenzene solvents, and the Stille coupling reaction lasted for 24 h in order to ensure that the degree of polymerization of the target product, DPP-2S-Se, was sufficiently large. The choice of chlorobenzene as a solvent is partly due to its high boiling point and partly to the comparatively high solubility of the polymer in it. As the degree of polymerization increases, the molecular weight rises, which leads to a rapid decrease in solubility in normal organic solvents. The purification of polymers relies primarily on Soxhlet extraction, specifically, taking advantage of the differences in the solubility of different components in various solvents. The sequential use of hexane, acetone, methanol, and ethyl acetate effectively eliminates oligomers and small-molecule materials from the reaction system. Polymers and oligomers with shorter chain lengths dissolve in acetone or hexane and present a blue–violet color, while target polymers with longer chain lengths dissolve only in chloroform and present a dark green color. Copolymer PDPP-2S-Se was filtered under reduced pressure into a dark green film state, as shown in the figure below.

**Figure 3:**
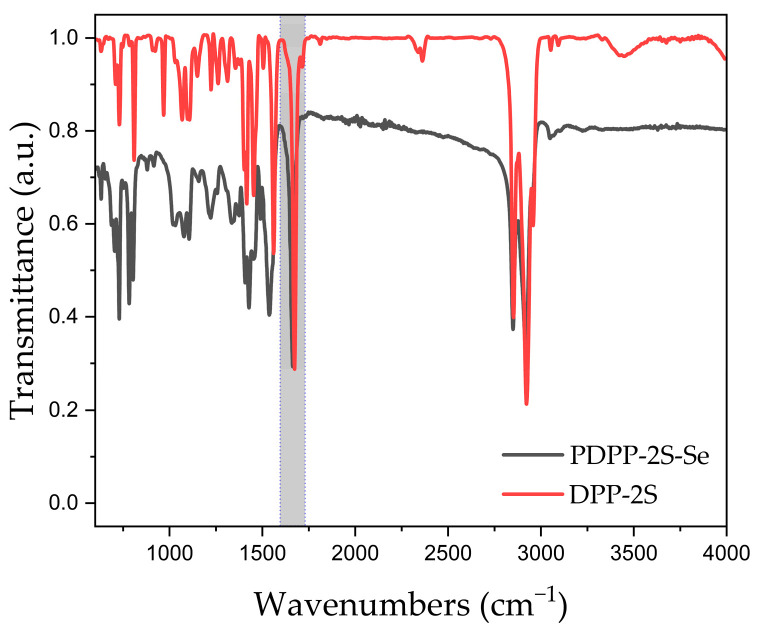
FT-IR spectrum of PDPP-2S-2Se polymer, the grey part belongs to the carbonyl peak.

The weight-average molecular weight (Mw) and the number-average molecular weight (Mn) for PDPP-2S-Se were 39.5 kDa and 32.2 kDa, respectively, which were measured using high-temperature gel permeation chromatography. The solvent flow phase chosen here was 1,3,5-trichlorobenzene to fully ensure the solubility of the material (Figure S1 in the Supplementary Information). The polymer-dispersion index (PDI) of the polymer was calculated to be 1.2, counting on the ratio of Mw to Mn, and the narrow PDI proves that the polymer is of great purity and the individual chain lengths are relatively homogeneous. In contrast to most of the reported DPP-based polymers with broader dispersions, mostly in the range of 2–3, the effect of reprecipitation purification of the monomers and suitable catalyst-to-ligand ratios on the purity of the polymers was demonstrated [25]. The degree of polymerization of each main chain was roughly estimated to be 28–29 repeat units based on the value of Mn. Elemental analysis of the polymers showed that the contents of the four elements, C, H, N, and O, were within 0.5% deviation from the theoretical values (Table 1). This further indicates the high purity of the material, which does not contain catalyst and ligand components. Figure 3 presents the Fourier transform infrared (FT-IR) comparison of the monomer DPP-2S and the polymer PDPP-2S-Se. There is a small migration of the carbonyl functional group position from 1673 cm^−1^ to 1662 cm^−1^ [26].

**Figure 4:**
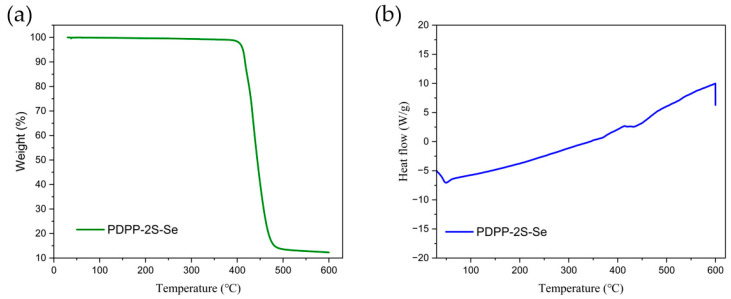
(**a**) TGA and (**b**) DSC analysis for PDPP-2S-Se polymer.

The polymer undergoes significant decomposition up to 400 °C, and shows a 10% weight loss at 416 °C (Figure 4a). The polymer does not show stages of decomposition, which is related to the uniform distribution of the polymer chain length. The polymer is almost completely decomposed in the 450–500 °C range. The results of the DSC measurements were in agreement with the TGA results in that there was no significant phase transition in the polymer until 413 °C, which demonstrated the good thermal stability of the polymer (Figure 4b). The polymer was examined for solubility and showed good solubility in chlorinated solvents. The solubility in dichloromethane, chloroform, and chlorobenzene increases gradually at room temperature. Upon a warming to 50 °C, the solubility in chlorobenzene rises significantly to 10 mg/mL. On the other hand, it is poorly soluble in non-chlorinated solvents such as ethanol, hexane, ethyl acetate, and tetrahydrofuran. The polymer solid remains almost insoluble even when heated. Although the use of ultrasound can assist its solubility in tetrahydrofuran, it is not necessary in view of the possibility of main chain breakage. In the subsequent device preparation, chlorobenzene was chosen as the solvent to prepare the organic semiconductor thin-film layer.

### 3.2. Density Functional Theory Calculations

**Figure 5:**
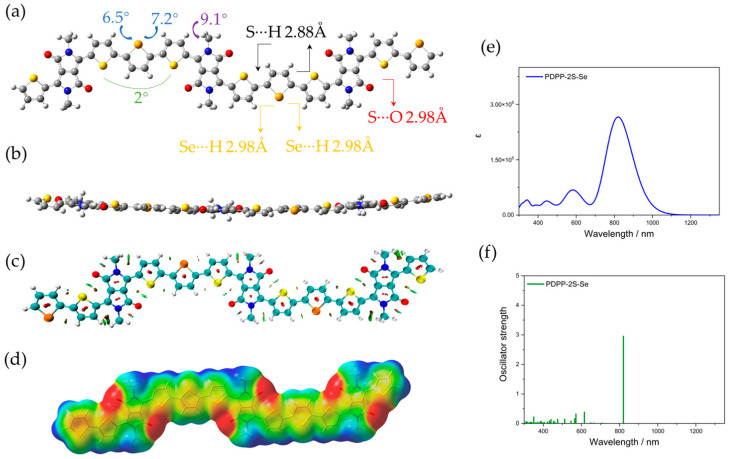
Top view of the optimized conjugated backbone conformation of the methyl-substituted trimer of the polymer (**a**); side view (**b**); (**c**) intramolecular non-covalent interactions; (**d**) electrostatic potential surface; (**e**) calculated absorption spectra of the trimer; (**f**) calculated Oscillator strength spectra of the trimer.

Since polymers are difficult to grow into crystals, Density Functional Theory (DFT) simulations are used here to obtain detailed information about the structure of the material. In particular, the London dispersion effects are incorporated to further obtain precisely optimized structures, although this increases the computational cost and time slightly [27,28]. The conjugation length of polymers is relatively long, and in order to maximally balance the difficulty of the calculations with the accuracy of the simulations, a trimer with three repeating units is used here as the model of study [29]. Further replacing the complex long chains on the side chains with shorter methyl groups can significantly reduce the computational time, and the non-conjugated chain has almost no effect on the energy levels of the material [30]. Table 2 lists the theoretical oscillator strengths, HOMO, LUMO, and HOMO−LUMO gap (E_c_) values of the trimer calculated according to the Gaussian 16 program of B3LYP-D3/def2tzvp [31,32,33,34]. Details of the optimized lowest energy configuration’s geometry and spatial coordinates are presented in the SI. DPP, consisting of two five-membered aromatic rings, exhibits a planar and rigid structure (Figure 5a,b). The thiophene on both sides is connected to the DPP by a single bond, where the S atom and the O atom of the carbonyl group present a weak S...O interaction, as their distance is only 2.98 Å. The planes of thiophene and DPP present a dihedral angle of 9.1°, and selenophene with two adjacent thiophenes exhibits dihedral angles of 6.5° and 7.2°, respectively. It is noteworthy that the thiophene linked by the bridge group selenophene is almost coplanar, with a dihedral angle of only 2°. The distance of Se from the H on the neighboring thiophene and the distance of the S atom on the thiophene from the H on the neighboring selenophene are 2.98 Å and 2.88 Å, respectively. There is a definite symmetry in the conformational existence between the three aromatic rings, where the S...H and Se...H distances are all less than the radial value of van der Waals forces. As shown in Figure 5c, non-covalent interaction analyses were carried out to observe and distinguish non-covalent interactions within the molecule [35]. Interestingly, according to theoretical simulations of non-covalent interaction calculations, the hydrogen atom on the methyl group of the nitrogen position also has a weak van der Waals force on the hydrogen atom on the thiophene, and its repulsive effect is not obvious. The theoretical simulation of the trimer electrostatic potential surface is illustrated in Figure 5d. Further DFT calculations were performed to simulate the UV-Vis absorption spectra, as shown in Figure 5e,f [36]. The calculation showed that the main absorption peak of the trimer was 825 nm and the onset absorption peak was 995 nm, corresponding to an energy gap of 1.25 eV. The molar absorption coefficient (ε) and oscillator strength (f_osc_) of the polymer were found to be 265,605 and 2.97, respectively. This is consistent with the fact that linearly conjugated polymers generally have a larger f_osc_ than cross-conjugation [37].

**Figure 6:**
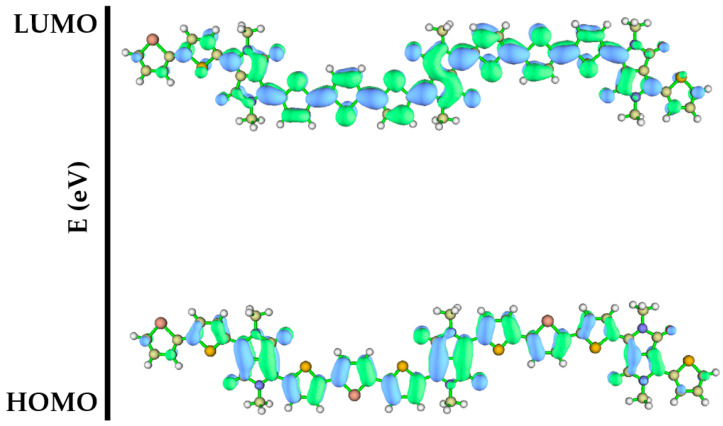
HOMO and LUMO orbital diagram of the trimer.

The calculated E_HOMO_ and E_LUMO_ values were −4.94 eV and −3.19 eV, respectively, and their electron cloud orbital maps are included in Figure 6. HOMO and LUMO are fully delocalized on trimers and π-conjugated bridges based on the D-A structure. HOMO predominantly occupies double bonds along the long axis of the molecule, whereas LUMO is predominantly distributed on single bonds perpendicular to the long axis. The material possesses a high HOMO energy level, which is matched to the working electrode, suggesting that this material has potential for p-type materials, that is, hole transport functions. The difference based on HOMO and LUMO shows a small energy-level gap of 1.75 eV for this compound. Figure S2 in the SI shows the corresponding HOMO+1 (−5.13 eV), HOMO+2 (−5.32 eV), and HOMO+3 (−5.99 eV), along with the LUMO+1 (−2.95 eV), LUMO+2 (−2.71 eV), and LUMO+3 (−2.19 eV) of the trimer.

### 3.3. Photophysical and Electrochemical Properties

**Figure 7:**
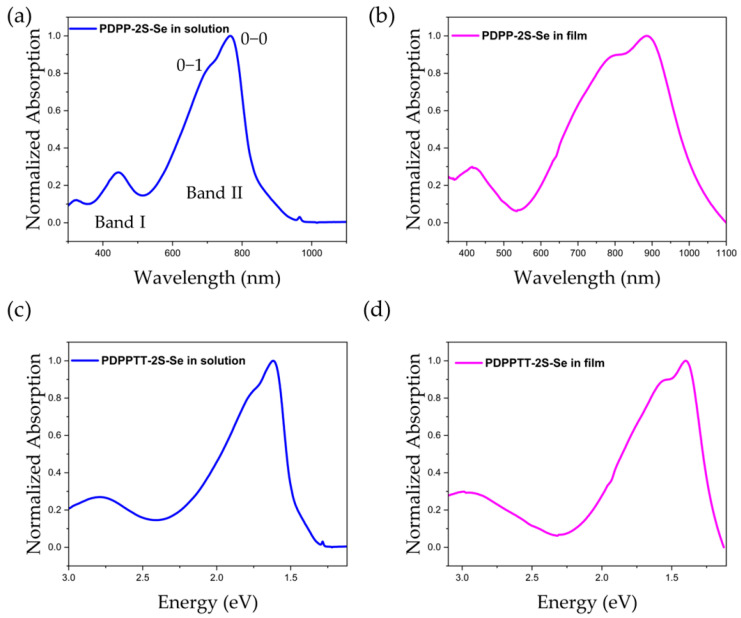
Normalized UV-Vis spectrum of PDPP-2S-Se in chloroform solution (**a**,**c**) and in thin film (**b**,**d**).

The UV-Vis optical absorption spectra of PDPP-2S-Se in solution and in the solid state (thin films) are shown in Figure 7 below, and the corresponding data are presented in Table 3. The concentration of the solution phase was approximately 0.5 mg/mL and chloroform was used as the solvent. The thin film was prepared by taking two drops of the solution onto a quartz plate and waiting for the solvent to evaporate slowly. There are two absorption bands in the solution with wavelength regions of 350−510 nm and 600−900 nm, respectively. High-energy absorption bands where the polymer is absorbing at low wavelengths are attributed to the π−π* transition. Absorption at long wavelengths (low-energy absorption) is due to the intramolecular charge transfer transition (Figure 7a). The main absorption peak (λ_max_) in band I is 445 nm, whereas the main absorption peaks in band II are 718 nm and 765 nm, corresponding to 0−1 and 0−0 peaks, respectively, with a ratio of 0.85. From the solution to the film, the main absorption peaks in band II are red-shifted to 799 nm and 884 nm with an elevated intensity ratio of 0.90 (Figure 7b). These changes are associated with solid-state stacking with better coplanar backbone and inter-polymer π−π stacking distance. The optical bandgap of the material is 1.45 eV and 1.22 eV for the solution (E_g_^soln^) and film (E_g_^film^), respectively, which is determined at the onset of UV-visible absorption at 855 nm and 1020 nm, respectively (Figure 7c,d). The curves of UV absorption match to some extent the results of the absorption curves calculated using DFT simulations.

**Figure 8:**
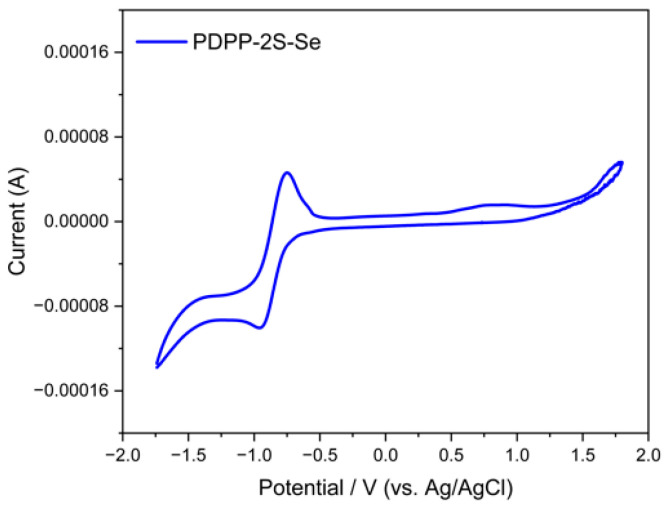
CV of PDPP-2S-Se film in the acetonitrile solution.

The redox potential of the PDPP-2S-Se polymer was investigated under a nitrogen atmosphere using a three-electrode system with cyclic voltammetry (CV). The polymer film was prepared by dissolving it in chlorobenzene; a solution of 1 mg/mL was first prepared before drop-casting it on the surface of the glassy carbon electrode and gently blowing it using a wash ball, causing the chlorobenzene solvent to slowly evaporate. The reduction attributed to the carbonyl group on the main chain shows a reversible reduction process with a reduction peak of −0.95 V (Figure 8). The ferrocene reference, used as an internal standard for electrochemical measurements, was measured at the same condition as the 0.39 V. The HOMO/LUMO energy levels of the material are −5.29 eV and −3.79 eV, respectively, which were calculated from the onset oxidation potential and onset reduction potential (Table 3). The energy level difference obtained from the calculation based on electrochemical tests is 1.50 eV, which is relatively comparable to the energy level gap calculated from the DFT.

### 3.4. OFET Performance

**Figure 9:**
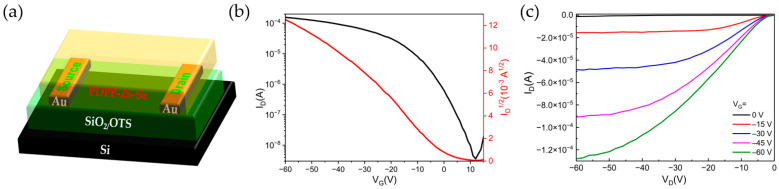
(**a**) OFET with BGBC architectures; (**b**) transport characteristics of OFET measured under optimized annealing temperature (220 °C); (**c**) output characteristics of PDPP-2S-2Se-based device.

To investigate the charge transport properties of the material, we fabricated PDPP-2S-Se-based OFET devices with a BGBC architecture (Figure 9a). Considering that its HOMO level matches that of the gold electrode (Au, approximately −5.00 eV), Au was chosen as the contact electrode with the semiconductor layer. Source-drain Au electrodes were generated with photolithography. A layer was deposited on the OTS-treated substrate by spin-coating in thermal chlorobenzene to form the polymer semiconductor film (6 mg/mL). Subsequently, in order to anneal the OSC films, the substrate was laid on a hot plate at a temperature of 220 °C for a heating time of 30 min and then slowly cooled to room temperature. The field-effect hole mobility (µ) was determined from the equation below [38]:

I_DS_ = µ (W/2L) C_i_ (V_GS_ − V_TH_)^2^

where W and L are the width and length of the channel, respectively; V_GS_ is the gate voltage and V_TH_ is the threshold voltage; and Ci is the capacitance per unit area of the gate dielectric layer.

Table 4 summarizes the key parameters of the device with the hole mobility, threshold voltage, and on/off current ratios extracted from the transport characteristic curve. It is clear from Figure 9b that the PDPP-2S-Se-based OFET device displays p-type unipolar transfer characteristics. The maximum dominant carrier value is 0.59 cm^2^ V^−1^ s^−1^. The average hole mobility obtained based on eight device measurements is 0.39 cm^2^ V^−1^ s^−1^. The maximum difference obtained between the on and off states is greater than 10^4^, which indicates that the material has good switching characteristics. The large on/off current ratio facilitates the practical application of OFETs in high-speed, low-power, and high-reliability circuits. The output behavior of the PDPP-2S-Se-based device is shown in Figure 9c.

## 4. Discussion

**Figure 10:**
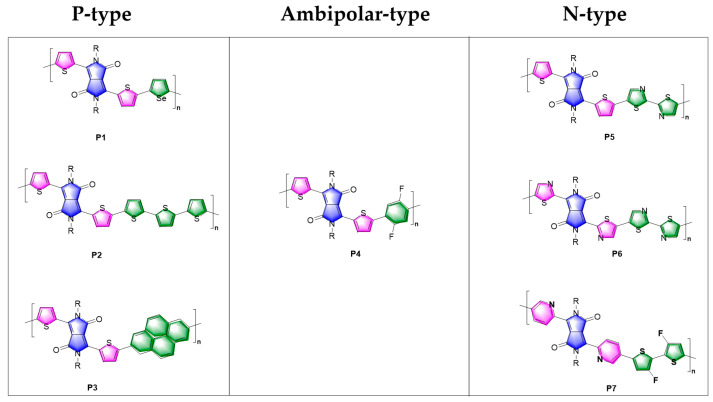
Molecular structures of typical polymers based on three materials of P-type, Ambipolar and N-type.

Recently, we reported the polymer material P5 based on DPP as the parent core and showed good electron mobility. For the structural comparison of P5 with P1, it may be possible to explore the relationship between molecular composition and macroscopic properties (Figure 10). The DPP represented by the blue part consists of two five-membered aromatic rings with a planar structure, and its inner molecule contains carbonyl groups that give it strong electron-withdrawing properties. The currently reported DPPs cannot be polymerized directly with the donor or acceptor using hydrocarbon activation or palladium-catalyzed coupling, but must be polymerized indirectly by linking two thiophene rings or other aromatic rings. As shown in the structure of P1, the introduction of thiophene facilitates the Stille coupling reaction with tin-containing monomers on the one hand, and on the other hand reduces the ability of the overall molecule to act as an electron acceptor and improves its potential to serve as an electron donor. In other words, this provides a good opportunity to change the energy levels and carrier behavior of the material through chemical modification strategies. In contrast to P5, a structure with multiple acceptors, polymer P1 contains various electron-donating groups such as thiophene and selenophene. Similarly, polymers P2 and P3 exhibit typical p-type material properties by introducing more electron-donor groups, with hole mobilities of 0.89 cm^2^ V^−1^ s^−1^ and 0.08 cm^2^ V^−1^ s^−1^, respectively [39,40]. The weak electron-donating ability of the benzene ring compared with thiophene and the further introduction of strongly electronegative F atoms into the P4 polymer significantly balances the strength of donors and acceptors throughout the system, with the material displaying a hole mobility of 0.03 cm^2^ V^−1^ s^−1^ and an electron mobility of 0.12 cm^2^ V^−1^ s^−1^ [41]. In contrast to P5, polymers P6 and P7 replace the thiophene, which is directly attached to the DPP, with thiazole and pyridine rings, respectively, which are strong electron-withdrawing moieties. The materials based on P6 and P7 show typical characteristics of n-type materials with electron mobilities of 0.07 cm^2^ V^−1^ s^−1^ and 0.20 cm^2^ V^−1^ s^−1^, respectively [42,43].

## 5. Conclusions

In summary, we have prepared polymeric materials with a donor-acceptor structure using the Stille coupling polymerization reaction, which shows a large molecular weight and a narrow dispersion. The DFT results show that the polymer is well-planar and the HOMO energy level matches the working electrode. OFET devices based on this polymer show high hole mobility and a sensitive switching ratio. Related work on the preparation of the polymer on a large scale (gram level) and further improvement of the carrier mobility remains on-going.

## Figures and Tables

**Table 1:** Elemental analysis for PDPP-2S-Se polymers.

	C	H	N	S
	(%)	(%)	(%)	(%)
theoretical value ^1^	71.89	9.69	2.54	5.81
1st experimental value	71.42	9.60	2.28	6.20
2nd experimental value	71.67	9.68	2.31	6.21
average value	71.54	9.64	2.30	6.20

^1^ The calculated theoretical values are from simulations from the repeating units of C_66_H_106_N_2_O_2_S_2_Se.

**Table 2:** Theoretically computed results for the oscillator strengths, frontier orbital energy levels of polymer.

	ε (cm^−1^ M^−1^)	f_osc_	λ_max_ ^caclu^ (nm)	λ_onset_ ^caclu^ (nm)	E_opt_ ^2^ (eV)	HOMO (eV)	LUMO (eV)	E_c_ ^3^ (eV)
PDPP-2S-Se ^1^	265605	2.97	825	995	1.25	−4.94	−3.19	1.75

^1^ Calculations come from the methyl-substituted trimers of PDPP-2S-Se; ^2^ calculations come from 1240/λ_onset_; ^3^ calculations come from HOMO—LUMO.

**Table 3:** Optical and electrochemical characteristics of the polymer PDPP-2S-Se.

	λ_max_^soln^ (nm)	λ_max_^film^ (nm)	E_g_^soln^ (eV) ^1^	E_g_^film^ (eV) ^1^	LUMO (eV) ^2^	HOMO (eV) ^3^	E_g_^cv^ (eV) ^4^
PDPP-2S-Se	765	884	1.45	1.22	−3.79	−5.29	1.50

^1^ E_g_^opt^ = 1240/λ_onset_; ^2^ E_LUMO_ = −4.80 eV − [(E_red_^onset^) − E_1/2_(ferrocene)]; ^3^ E_HOMO_ = −4.80 eV − [(E_ox_^onset^) − E_1/2_(ferrocene)]; ^4^ E_g_^cv^ = E_HOMO_ − E_LUMO_.

**Table 4:** Hole transport properties of PDPP-2S-Se-based OFET device.

	Rotation Speed (mm/s)	Annealing Temperature (°C)	Hole Mobilities ^1^ (cm^2^/(V s))	Max Mobilities (cm^2^/(V s))	Threshold Voltages (V)	I_ON/OFF_
PDPPTT-2Tz	2000	220	0.39	0.59	0.2	10^4^–10^5^

^1^ Based on eight OFET devices for each condition.

## Data Availability

Not applicable.

## Supplementary Materials

The following supporting information can be downloaded at: https://www.mdpi.com/article/10.3390/polym15183713/s1, Figure S1: GPC data (cumulative percent curves and molecular weight distribution) of the polymer PDPP-2S-Se; Figure S2: Calculated HOMO+1, LUMO+1, HOMO+2, LOMO+2, HOMO+3, and LOMO+3 for the trimer; Figure S3: Optimized geometry; Table S1: Spatial coordinates.
