# Supplementary material for: Donor-Acceptor-Based Organic Polymer Semiconductor Materials to Achieve High Hole Mobility in Organic Field-Effect Transistors
Source: Polymers (Basel). 2023 Sep 9;15(18):3713. doi: 10.3390/polym15183713 (PMC10538171; doi:10.3390/polym15183713)
Supplement: Supplementary file 1 [file polymers-15-03713-s001.zip › polymers-2594152-supplementary.pdf]

Supplementary Information

Synthesis of DPP-2S-C_{10} monomer: To a solution of DPP-2S (1.00 g, 3.33 mmol) in anhydrous dimethylformamide (DMF, 30 ml) was passed argon, followed by the addition of potassium carbonate (K_2CO_3, 1.38 g, 10.00 mmol, 3.00 eq) in batches over a period of half an hour. Next, 11-(3-bromopropyl) henicosane (3.48 g, 8.33 mmol, 2.50 eq) was added dropwise to the flask using a syringe. The mixture was stirred at 100 °C overnight. It was extracted with dichloromethane (DCM), then washed with water and brine and dried with Na_2SO_4. After removal of the solvent under reduced pressure, the residue was purified using silica gel chromatography with the eluent (petroleum ether: DCM = 4:1) to afford a red, solid brick (2.20 g, 67.9 %). δ ^1H NMR (400 MHz, Chloroform-d) δ 8.92 (d, J = 3.8 Hz, 2H), 7.62 (d, J = 4.9 Hz, 2H), 7.30 – 7.26 (t, J = 3.6 Hz, 2H), 4.03 (t, J = 7.9 Hz, 4H), 1.77 – 1.62 (m, 4H), 1.36 – 1.20 (m, 78H), 0.89 (t, J = 6.7 Hz, 12H). ^{13}C NMR (75 MHz, Chloroform-d) δ 161.35, 140.02, 135.28, 130.60, 129.82, 128.63, 107.73, 42.58, 37.17, 33.56, 31.95, 30.52, 30.11, 29.73, 29.68, 29.38, 27.08, 26.71, 22.71, 14.15. Mass: $[M^+H]^+$ calculated for $C_{62}H_{104}N_2O_2S_2^+$: 972.7539; found: 972.7539.

Synthesis of DPP-2S-Br monomer: Argon was passed to a solution of DPP-2S-C_{10} (1.00 g, 1.03 mmol) in chloroform ($CHCl_3$, 20 ml), and N-Bromosuccinimide (NBS, 0.37 g, 2.11 mmol, 2.05 equiv) was added. The mixture was stirred at 60 °C for half an hour. The mixture was then rapidly extracted with dichloromethane in order to avoid the formation of by-products. Water and brine were followed by washing and drying with Na_2SO_4. After removal of the solvent under reduced pressure, the residue was purified using silica gel chromatography with eluent (petroleum ether: DCM = 5:1) to obtain a crimson powder (1.10 g, 94.8 %). ^1H NMR (300 MHz, Chloroform-d) δ 8.68 (d, J = 4.2 Hz, 2H), 7.25 (d, J = 4.2 Hz, 2H), 3.94 (t, J = 7.8 Hz, 4H), 1.77 – 1.63 (m, 4H), 1.44 – 1.20 (m, 78H), 0.89 (t, J = 6.5 Hz, 12H). ^{13}C NMR (75 MHz, Chloroform-d) δ 161.42, 139.42, 135.32, 131.44, 131.20, 118.96, 108.05, 46.37, 37.78, 31.94, 31.90, 31.20, 30.00, 29.66, 29.57, 29.51, 29.38, 29.30, 26.20, 22.71, 22.68, 14.13. Mass: $[M^+H]^+$ calcd for $C_{54}H_{87}Br_2N_2O_2S_2^+$: 1128.5749; found: 1128.5742.

Synthesis of PDPP-2S-Se polymer: DPP-2S-Br (200 mg, 0.18 mmol, 1.0 eq), 2,5-bis(trimethylstannyl) selenophene (80.92 mg, 0.18 mmol, 1.0 eq), and triethyl phosphite (P(o-tol)$_3$, 4.32 mg, 14.18 μmol) were dissolved in dry chlorobenzene (9 ml). Argon was passed into the Schlenk tube, and 10 minutes later tris(dibenzylideneacetone)dipalladium catalyst ([Pd_2(dba)$_3$], 3.24 mg, 3.54 μmol) was rapidly added. The reaction was stirred at 130 °C. After approximately fifteen minutes, the color of the solution changed from red to purple and finally stabilized to dark green. The polymerization was completed after 24 hours. Sodium diethyldithiocarbamate trihydrate was added to remove the [Pd_2(dba)$_3$]. The mixture was dropped into methanol (200 mL) and filtered to afford a black precipitated product. Purification with Soxhlet extraction using n-hexane (10 h), methanol (10 h), ethyl acetate (10 h), and acetone (10 h) gave blue, light red, cyan, and purple impurities, respectively. Finally, the target polymer was obtained using the chloroform phase and its fractions were dissolved in chloroform, showing dark green color. The fractions were evaporated and concentrated, precipitated into methanol (200 mL), and filtered to obtain the polymeric material in the form of dark-colored film, which was further dried under vacuum for 3 h (90 °C) to obtain PDPP-2S-Se (183.5 mg, 91.0 %).

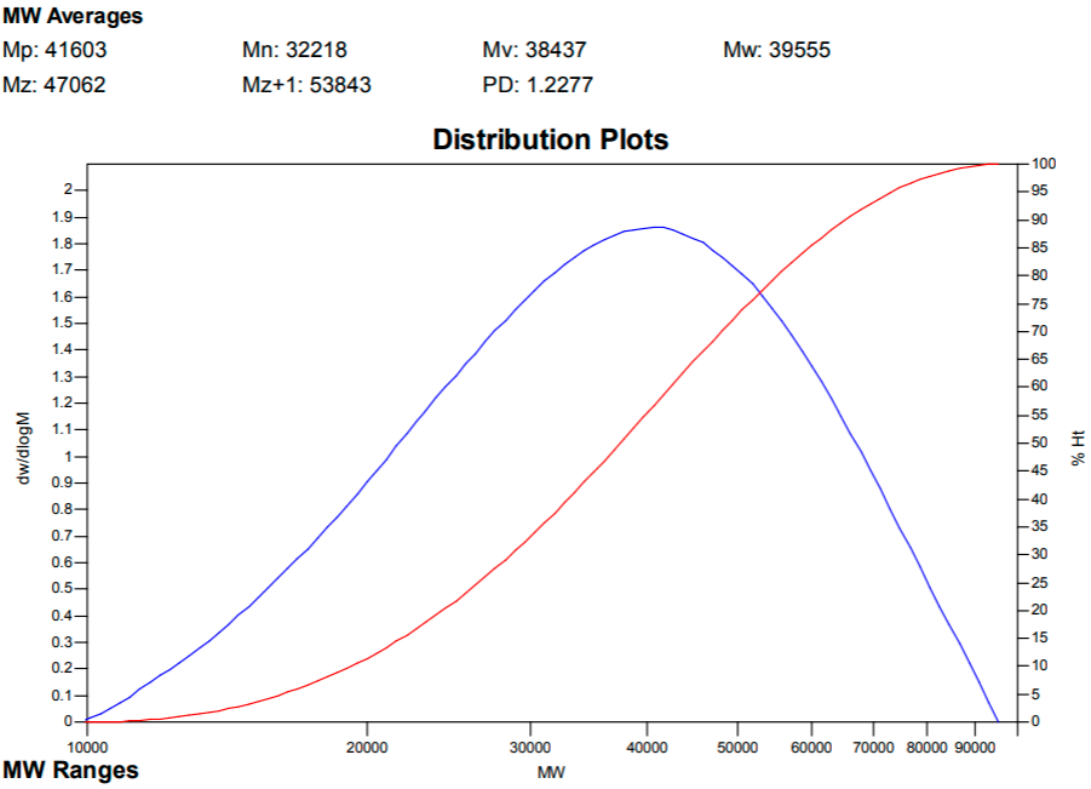

Figure S1. GPC data (cumulative percent curves and molecular weight distribution) for PDPP-2S-Se polymer.

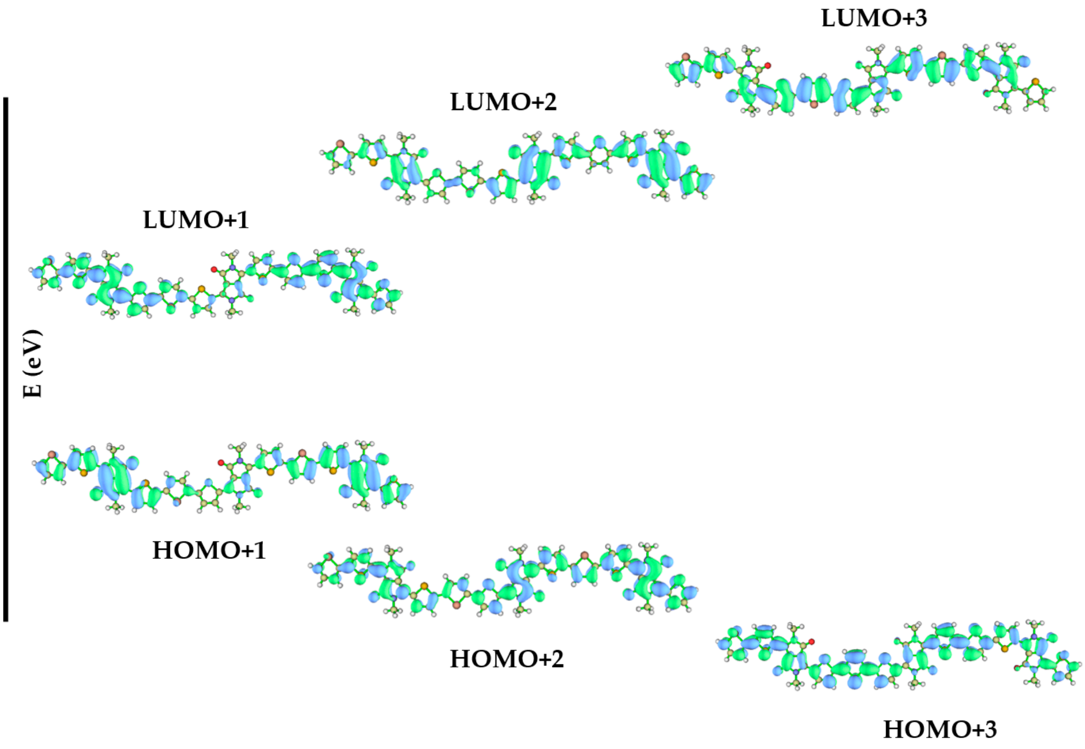

Figure S2. Calculated HOMO+1, LUMO+1, HOMO+2, LOMO+2, HOMO+3, and LOMO+3 for the trimer.

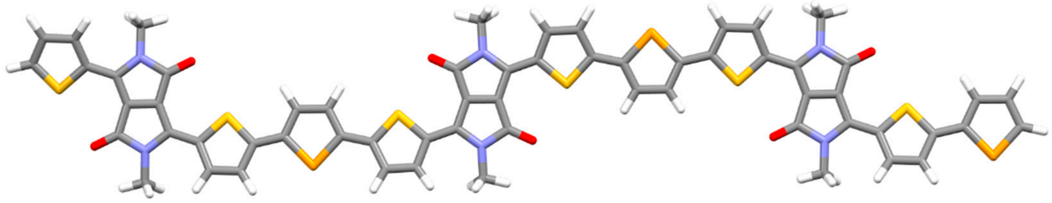

Figure S3. Optimized geometry.

Table S1. Spatial coordinates.

**C**	**18.70893**	**1.25289**	**0.40198**
**C**	**18.2139**	**−0.04217**	**0.43431**
**C**	**16.80022**	**−0.01184**	**0.30342**
**C**	**16.38337**	**1.36714**	**0.21192**
**N**	**17.6139**	**2.1011**	**0.27263**
**C**	**18.63652**	**−1.42244**	**0.4785**
**N**	**17.41365**	**−2.15892**	**0.34513**
**C**	**16.31343**	**−1.31098**	**0.23704**
**O**	**15.29448**	**1.90672**	**0.12641**
**O**	**19.72102**	**−1.9582**	**0.61534**
**C**	**14.96389**	**−1.75159**	**0.07314**
**C**	**20.07152**	**1.6979**	**0.46613**
**C**	**14.46206**	**−3.01909**	**−0.19609**
**C**	**13.069**	**−3.05971**	**−0.30754**
**C**	**12.46548**	**−1.82774**	**−0.12324**
**S**	**13.65081**	**−0.60259**	**0.18398**
**C**	**20.61643**	**2.93656**	**0.16551**
**C**	**22.01337**	**2.98909**	**0.33649**
**C**	**22.53033**	**1.79781**	**0.77161**
**S**	**21.33279**	**0.59523**	**0.9578**
**C**	**17.43789**	**−3.59999**	**0.45405**
**C**	**11.07138**	**−1.50574**	**−0.16869**
**Se**	**9.75141**	**−2.85214**	**−0.32155**
**C**	**8.45739**	**−1.47226**	**−0.30097**
**C**	**9.07236**	**−0.24833**	**−0.18103**
**C**	**10.47892**	**−0.26639**	**−0.11004**
**C**	**7.05833**	**−1.75851**	**−0.39677**
**C**	**17.57906**	**3.54561**	**0.32827**
**C**	**6.44575**	**−2.97282**	**−0.65443**
**C**	**5.05041**	**−2.8975**	**−0.68154**
**C**	**4.55451**	**−1.61982**	**−0.44765**
**S**	**5.87923**	**−0.50634**	**−0.19124**
**C**	**3.20743**	**−1.14694**	**−0.41009**
**N**	**2.07942**	**−1.96326**	**−0.45528**
**C**	**0.87041**	**−1.19551**	**−0.41729**
**C**	**1.3306**	**0.17124**	**−0.35212**
**C**	**2.74798**	**0.16372**	**−0.33729**
**C**	**0.87189**	**1.48425**	**−0.34802**
**N**	**2.00008**	**2.30095**	**−0.31523**
**C**	**3.20847**	**1.53153**	**−0.30226**
**O**	**4.31582**	**2.03724**	**−0.2533**

O	−0.23733	−1.70268	−0.42544
C	−0.47431	1.95949	−0.38269
C	2.07458	3.73971	−0.20013
C	2.00339	−3.40643	−0.43722
S	−1.8034	0.83231	−0.22858
C	−2.97889	2.09613	−0.37526
C	−2.36179	3.32448	−0.53918
C	−0.96621	3.25019	−0.54193
C	−4.37933	1.80528	−0.32763
C	−4.99574	0.57655	−0.29603
C	−6.4034	0.5907	−0.25429
C	−6.9958	1.83162	−0.24983
Se	−5.67399	3.18439	−0.29216
C	−8.39062	2.15063	−0.21446
S	−9.58146	0.90845	−0.01407
C	−10.8934	2.063	−0.07889
C	−10.3862	3.3453	−0.25357
C	−8.99118	3.39225	−0.33103
C	−12.2454	1.61395	0.02728
C	−12.7327	0.31252	0.01125
C	−14.1474	0.33444	0.11217
C	−14.5734	1.70954	0.21964
N	−13.3502	2.45319	0.15548
C	−12.3096	−1.06004	−0.13563
N	−13.537	−1.79934	−0.13352
C	−14.6399	−0.96163	0.01216
O	−11.2167	−1.59051	−0.22679
O	−15.663	2.23736	0.35618
C	−13.3817	3.88718	0.33291
C	−13.496	−3.24394	−0.16305
C	−15.9977	−1.40673	0.03339
C	−16.5371	−2.65151	−0.26429
C	−17.931	−2.70195	−0.14877
C	−18.4936	−1.50198	0.24358
S	−17.2754	−0.29428	0.46675
C	−19.8813	−1.19305	0.45539
Se	−21.1945	−2.55287	0.54479
C	−22.4507	−1.1974	0.81007
C	−21.8695	0.0318	0.82006
C	−20.4625	0.03646	0.62628
H	15.07941	−3.88885	−0.33394
H	12.51516	−3.96056	−0.53089
H	20.04037	3.77262	−0.19043
H	22.61091	3.86774	0.14315
H	23.56134	1.56416	0.98265
H	16.74262	−3.9477	1.21812
H	17.20602	−4.08236	−0.49733
H	18.45269	−3.87026	0.73759
H	8.50566	0.67317	−0.15752
H	11.06368	0.64041	−0.02841
H	16.53653	3.82558	0.46203
H	18.16843	3.91747	1.16575
H	17.93898	3.9962	−0.59858

H	6.99658	−3.88551	−0.83268
H	4.42983	−3.75084	−0.88838
H	1.47942	4.09862	0.63972
H	3.12148	3.98043	−0.02935
H	1.75045	4.23595	−1.11664
H	0.95324	−3.65702	−0.30493
H	2.34709	−3.84125	−1.37749
H	2.58081	−3.82065	0.38945
H	−2.90937	4.24742	−0.66766
H	−0.34213	4.115	−0.67875
H	−4.4288	−0.3449	−0.31625
H	−6.9889	−0.31917	−0.24019
H	−11.0005	4.22294	−0.34708
H	−8.43341	4.30565	−0.48201
H	−14.4059	4.14214	0.59599
H	−12.7129	4.19859	1.1354
H	−13.1199	4.41487	−0.58609
H	−14.0599	−3.66913	0.66709
H	−13.8784	−3.64073	−1.10528
H	−12.4491	−3.52455	−0.07177
H	−15.952	−3.49577	−0.583
H	−18.516	−3.58476	−0.36433
H	−23.4946	−1.43423	0.93253
H	−22.4385	0.9407	0.96031
H	−19.8832	0.95005	0.59908
